# Compositional plaque progression in women and men with non-obstructive coronary artery disease

**DOI:** 10.1016/j.ijcha.2024.101352

**Published:** 2024-02-07

**Authors:** Caroline Annette Berge Hondros, Ingela Khan, Margrete Solvik, Silja Hanseth, Eva Kristine Ringdal Pedersen, Siren Hovland, Terje Hjalmar Larsen, Mai Tone Lønnebakken

**Affiliations:** a Department of Heart Disease, Haukeland University Hospital, Jonas Lies vei 65, 5021 Bergen, Norway; b Department of Clinical Science, University of Bergen, P.O. Box 7804, 5020 Bergen, Norway; c Department of Medicine, Haraldsplass Deaconess Hospital, Ulriksdal 8, 5009 Bergen, Norway; d Norwegian Registry of Invasive Cardiology, Haukeland University Hospital, Jonas Lies vei 65, 5021 Bergen, Norway; e Department of Biomedicine, University of Bergen, P.O. Box 7804, 5020 Bergen, Norway

**Keywords:** Non-obstructive coronary artery disease, Coronary computed tomography angiography, Sex differences, Plaque progression, Plaque composition

## Abstract

**Background:**

In coronary artery disease (CAD), plaque progression and plaque composition are associated with cardiovascular risk. Whether compositional plaque progression in non-obstructive CAD differs between women and men is less studied.

**Methods:**

We included 31 patients (42% women) with chronic non-obstructive CAD from the Norwegian Registry of Invasive Cardiology, undergoing serial coronary computed tomography angiography (CCTA) on clinical indication (median inter-scan interval 1.8 [1.5–2.2] years). We performed quantitative and qualitative plaque analysis of all coronary artery segments.

**Results:**

Women were older compared to men (65 ± 8 years vs. 55 ± 12 years, p = 0.019), while there was no difference in the prevalence of hypertension, diabetes, smoking or statin treatment between groups. At baseline, women had a higher total plaque burden, more calcified plaques, and less fibro-fatty and necrotic core plaques compared to men (all p < 0.05). During follow-up, men showed faster progression of fibro-fatty plaques (4.0 ± 5.4 % per year vs. −0.6 ± 3.1 % per year, p = 0.019) and a greater reduction of fibrous plaques (-7.3 ± 6.1 % per year vs. 2.1 ± 7.2 % per year, p = 0.003) compared to women even after age adjustment. At follow-up, total plaque burden remained higher in women compared to men (24.9 ± 3.3 % vs. 21.1 ± 2.6 %, p = 0.001), while men had an increase in fibro-fatty (21.2 ± 9.3 % vs. 28.6 ± 9.8 %, p = 0.004) and necrotic core plaques (5.6 ± 3.6 % vs. 10.8 ± 7.2 %, p = 0.006), and a decrease in fibrous plaques (69.0 ± 11.9 % vs. 54.7 ± 13.7 %, p < 0.001). Women’s plaque composition remained unaltered.

**Conclusion:**

In non-obstructive CAD, serial CCTA demonstrated a higher total plaque burden and a stable plaque composition in women, while men had a faster progression of unstable low-attenuating fibro-fatty plaques.

Clinical trial registration: ClinicalTrials.gov: Identifier NCT04009421.

## Graphical abstract

**Figure:**
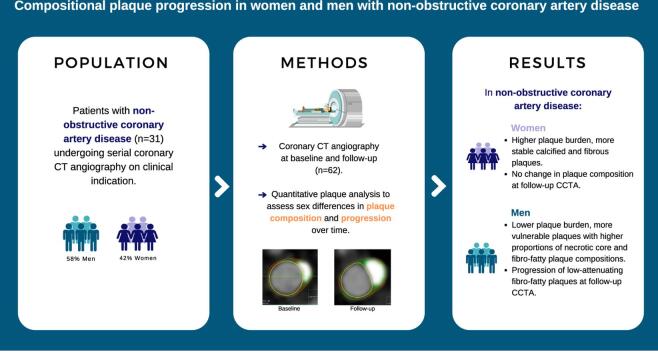

## 1 Introduction

The natural history of chronic non-obstructive coronary artery disease (CAD) is characterized by coronary artery plaque progression, plaque rupture or plaque erosion that may result in acute fatal or non-fatal cardiac events [1]. Recent coronary computed tomography angiography (CCTA) studies have identified both plaque progression and plaque composition as important predictors of developing plaque vulnerability that may lead to rupture and erosion. In particular, rapid plaque progression [2], low-attenuation fibro-fatty plaque [3] and microcalcifications [4] have been demonstrated to increase plaque vulnerability and risk of events. Furthermore, statin therapy has been associated with reduced plaque progression, transformation into more stable calcified plaques and reduced risk of events [5]. Yet there are currently no recommendations for serial CCTA to assess plaque progression in non-obstructive CAD [6]. Consequently, to improve risk stratification and individualize prevention and treatment in patients with non-obstructive CAD, identification of plaque progression to a more vulnerable plaque phenotype may be important.

Observed sex differences in CAD development, phenotype and event rate throughout life, suggest sex to be an important modifier of CAD progression [7]. In general, women are diagnosed with CAD at an older age and non-obstructive CAD is more common [8]. Even though women tend to exhibit lower plaque vulnerability compared to men, the menopausal transition is associated with increased plaque vulnerability in women, suggesting oestrogen to be cardioprotective [9]. However, non-obstructive CAD in symptomatic women is associated with increased cardiovascular (CV) risk [10]. Few studies have explored the impact of sex on compositional plaque progression by quantitative analysis of CCTA in patients with stable or suspected CAD, and the results are diverging [11], [12], [13], [14]. Furthermore, in the majority of these studies, women are often underrepresented. The understanding of sex as a modifier of CAD progression and plaque vulnerability in patients with chronic coronary syndrome is still limited. To address this knowledge gap, we hypothesized that sex modifies plaque progression and composition in non-obstructive CAD. Accordingly, we performed quantitative and qualitative plaque analysis to assess plaque progression and change in plaque composition over time in women and men with non-obstructive CAD and chronic coronary syndrome undergoing clinically indicated serial CCTA.

## 2 Methods

### 2.1 Study population and data source

Data of 38 individuals with serial CCTA examinations were retrieved from a customized module for CCTAs at the Norwegian Registry of Invasive Cardiology (NORIC), which is part of the Norwegian Cardiovascular Disease Registry [15]. All patients were diagnosed with non-obstructive CAD, defined as presence of atherosclerotic plaque with 1–49 % luminal diameter stenosis, at the baseline CCTA examination performed at Haukeland University Hospital between January 2016 and September 2019. All CCTA examinations were performed on clinical indication with >1 year inter-scan interval (median interval 1.8 [1.5–2.2] years). Furthermore, patients who experienced clinical events between examinations (n = 3), had uninterpretable CCTA scans with insufficient image quality (n = 1), or incomplete CCTA examinations (n = 3) were excluded from the study. The final study population included 31 individuals (13 women and 18 men). The study was approved by the Regional Ethical Committee for Medical and Health Research Ethics in Norway and conforms to the ethical guidelines of the 1975 Declaration of Helsinki. All patient data were anonymized; therefore, informed consent from patients was not required. The study was registered at https://www.clinicaltrials.gov with identifier NCT04009421.

### 2.2 Clinical characteristics

Available patient demographics and clinical characteristics were extracted from NORIC. Systemic arterial hypertension was defined as known hypertension or use of antihypertensive medication. Diabetes was defined as known diabetes. Glomerular filtration rate (GFR) was calculated using the CKD-EPI creatinine equation. History of smoking was defined as either current or former smoker. Clustering of common CV risk factors was considered present in individuals with ≥3 CV risk factors. Body mass index (BMI) was calculated as body weight divided by height in meters squared.

### 2.3 Coronary CT angiography acquisition

All coronary CT angiograms were performed on clinical indication, using a dual source Siemens scanner (SOMATOM Force 2 x 192-slice or SOMATOM Definition Flash 2 x 128-slice, Siemens, Germany). For coronary artery calcium scoring, a preliminary non-contrast electrocardiographic-triggered scan was acquired. Prior to the CCTA scans, patients received 0.4 mg sublingual nitroglycerine. Intravenous metoprolol 1 mg/ml (maximum 20 mg) was administered to patients with a heart rate >60 beats per minute until the heart rate was <60 beats per minute. All contrast enhanced CCTA examinations were performed with electrocardiographic triggered acquisition and intravenous iohexol 350 mg I/ml (Omnipaque®, GE Healthcare, Chicago, USA).

### 2.4 Coronary CT angiography assessment

Coronary artery calcium score, determined by the Agatston calcium score [16], and coronary artery segment involvement score (SIS) were extracted from the NORIC registry. The CCTA images were anonymized and analyzed using a semiautomated analysis software (QAngio CT Research Edition version 3.2.0.12, Medis medical imaging system, Leiden, The Netherlands) by experienced readers blinded to patient characteristics. The intra-observer reliability ICC for total plaque volume was 0.95 (95 % CI = 0.63–0.99), and for volumes of various plaque components: dense calcium 0.99 (95 % CI = 0.99–0.99), fibrous plaques 0.89 (0.53–0.97), fibro-fatty plaques 0.97 (0.91–0.99), and necrotic core 0.94 (0.82–0.98).

All coronary artery segments including side branches ≥ 2 mm in luminal diameter were included in the quantitative plaque analysis. Vessel and lumen contours of the coronary arteries were obtained automatically by the software and corrected manually when necessary. Plaque volume (mm^3^), vessel volume (mm^3^), and vessel length (mm) were obtained for all coronary artery segments. Plaque distribution was scored as segment involvement score (SIS), and extensive coronary artery disease defined as SIS > 4 [17]. In addition, visually identifiable coronary artery lesions were co-registered using anatomical landmarks such as distance from the ostium and subdivisions of the coronary vessels. At baseline CCTA, all lesions were non-obstructive, and at follow-up the lesions were classified as either obstructive (≥50 % lumen diameter stenosis) or non-obstructive. Plaque composition was quantified automatically based on predefined plaque attenuation measurements in Hounsfield Units (HU) and classified into calcified (>350 HU) or non-calcified plaque (≤350 HU), comprising necrotic core (−30–75 HU), fibro-fatty plaques (76–130 HU) and fibrous plaques (131–350 HU) [18].

To account for differences in vessel size between individuals, the plaque volume was presented as a percentage of vessel volume, as it has shown good diagnostic accuracy in the identification of CAD [19]. Percent plaque burden was defined as [(plaque volume/vessel volume) × 100] and reported in % [19]. To determine plaque development over time, annual change in plaque burden was estimated using change in plaque burden divided by the interval between CCTA examinations in years [5].

Furthermore, we evaluated qualitative plaque features for each lesion by assessing presence of at least two high-risk plaque (HRP) features, including spotty calcification, hypodense plaque and positive remodeling [5]. Spotty calcification was scored manually according to the presence of dense calcium measured <3 mm in any direction within the lesion [5]. The remodeling index was quantified automatically as the ratio between the vessel wall area with the greatest luminal stenosis at the site of the lesion, and the vessel wall area in a proximal normal coronary artery segment. A positive remodeling index was reported when the remodeling index was >1.10 [20].

### 2.5 Statistical analysis

Categorical variables are presented as percentages or numbers. Continuous variables are presented as mean (±SD) or median (interquartile range [IQR]) where appropriate. The Chi-square test or Fischer’s test was used to compare differences between categorical variables, and the Student’s *t*-test or the Mann-Whitney *U* test were used to compare differences between continuous variables.

To assess changes in coronary artery plaque characteristics between CCTA examinations, paired t-tests, Wilcoxon signed rank test, or McNemar’s test were performed where appropriate. Additionally, sex specific analyses were performed to assess potential sex differences in plaque progression by linear regression models adjusted for age. A two-sided *p* value < 0.05 was considered statistically significant. All data management and statistical analyses were performed using IBM SPSS Statistics 25 (IB Corporation, Armonk, NY, USA) and R Statistics 4.1.1. (The R foundation for statistical computing platform, Vienna, Austria).

## 3 Results

### 3.1 Clinical and coronary artery disease characteristics

**Fig. 1:**
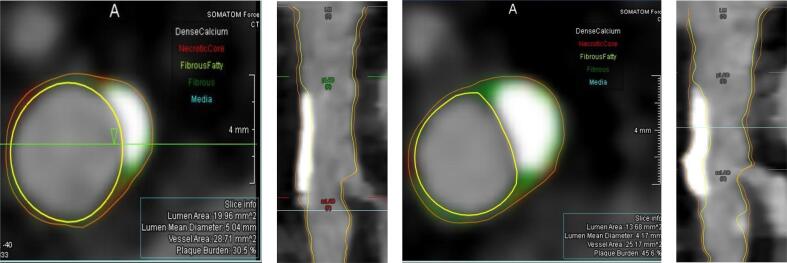
Left: CCTA of a lesion in the proximal left anterior descending (LAD) artery at baseline. Right: CCTA of the same lesion in the proximal LAD artery at follow-up.

A total of 62 coronary CT angiograms from 31 patients (42 % women) undergoing serial CCTA examinations on clinical indication were analysed (Fig. 1, Table 1). Women were older and had a lower GFR compared to men (Table 1). The prevalence of hypertension, diabetes, statin treatment and history of smoking at baseline are presented in Table 1. A trend towards higher prevalence of CV risk factors was observed in men (Table 1). Statin treatment did not differ between sexes at baseline or follow-up CCTA (Table 1). The baseline median coronary artery calcium score was 36.0 (6.5–108.3) and did not differ between groups (Table 1). In the total study population, the segment involvement score was 2.0 (1.0–4.0) and 13 % of patients had extensive non-obstructive CAD as defined by SIS > 4. A total of 26 % of lesions were distributed to the left main (LM) stem and the prevalence of HRP was high (68 %), without any observed sex differences.

**Table 1:** Baseline clinical characteristics of the study population.

	Total population	Women	Men	*p*-value
	N = 31	N = 13	N = 18	
Age, years	59.3 ± 11.6	64.9 ± 8.4	55.2 ± 12.2	**0.019**
Hypertension, %	39	23	50	0.129
Diabetes, %	13	8	17	0.621
GFR ± SD, mL/min/1.73 m^2^	76.7 ± 14.2	75.8 ± 19.5	94.6 ± 16.3	**0.007**
Statin treatment, %				
Statin treatment at baseline	39	39	39	0.981
Statin treatment at follow-up	57	50	61	0.547
History of smoking, %	81	77	83	0.676
Clustering of CV risk factors, %	17	8	25	0.343
BMI, kg/m^2^	27.2 ± 4.4	26.0 ± 5.3	28.0 ± 3.6	0.235
Calcium score, HU	36.0 (6.5–108.3)	34.0 (6.8–141.5)	36.0 (5.8–108.3)	0.687
CCTA interval, years	1.8 (1.5–2.2)	2.0 (1.6–2.7)	1.7 (1.4–2.2)	0.28

Numbers are n (%), mean ± standard deviation or median (interquartile range) where appropriate.

BMI: body mass index; CCTA: coronary computed tomography angiography; CV: cardiovascular; GFR: glomerular filtration rate.

Bold indicates statistically significant p-values defined as p < 0.05.

### 3.2 Sex differences in plaque characteristics by CCTA

The SIS, and the prevalence of extensive and obstructive CAD at follow-up did not differ between sexes (Table 2). The prevalence of qualitatively assessed HRP, including spotty calcification, hypodense plaque, and positive remodelling, remained high in both men and women at follow-up (Table 2). However, women had a larger quantitatively assessed total plaque burden, corrected for vessel volume, compared to men, particularly in the proximal LAD and circumflex artery (CX) at baseline and at follow-up (Table 2).

**Table 2:** Baseline and follow-up CCTA characteristics in women and men.

	Baseline		p-value between women vs. men	Follow-up		p-value between women vs. men
	Women	Men		Women	Men	
	n = 13	n = 18		n = 13	n = 18	
Segment involvement score	2.0 (2.0–4.0)	2.5 (1.0–4.0)	0.653	2.0 (2.0–4.0)	3.0 (2.0–4.25)	0.625
Extensive CAD (SIS > 4), %	15	11	1	15	22	1
Obstructive CAD, %	0	0	–	8	6	1
High-risk plaque*, %	69	67	1	69	67	1

*Plaque burden, %*						
Total plaque burden	25.6 ± 4.6	21.4 ± 2.3	**0.008**	24.9 ± 3.3	21.1 ± 2.6	**0.001**
LM	22.5 ± 5.3	19.2 ± 4.7	0.082	21.0 ± 5.8	17.9 ± 4.3	0.102
Proximal LAD	32.2 ± 9.2	23.3 ± 4.1	**0.005**	33.1 ± 11.4	24.2 ± 6.1	**0.02**
Proximal CX	26.6 ± 6.6	20.8 ± 2.9	**0.009**	25.9 ± 6.5	20.3 ± 2.7	**0.011**
Proximal RCA	22.8 ± 4.5	21.2 ± 3.3	0.279	21.8 ± 3.5	19.6 ± 2.7	0.051

*Compositional plaque burden, %*						
Calcified	20.1 ± 19.4	3.7 ± 3.2	**0.01**	16.7 ± 12.5	5.5 ± 4.4	**0.008**
Fibrous	64.9 ± 13.0	69.0 ± 11.9	0.373	67.5 ± 15.2	54.7 ± 13.7	**0.02**
Fibro-fatty	11.8 ± 9.9	21.2 ± 9.3	**0.011**	11.4 ± 9.9	28.6 ± 9.8	**<0.001**
Necrotic core	2.8 ± 2.7	5.6 ± 3.6	**0.023**	3.9 ± 6.4	10.8 ± 7.2	**0.01**

Numbers are n (%), mean ± standard deviation or median (interquartile range) where appropriate.

CAD: coronary artery disease; CX: circumflex; LAD: left anterior descending; LM: left main; RCA: right coronary artery; SIS: segment involvement score.

Bold indicates statistically significant p-values defined as p < 0.05.

*High-risk plaque defined as lesions having ≥2 high-risk plaque features, including hypodense plaque, spotty calcification, or positive remodeling.

Additionally, the plaque composition at baseline and follow-up CCTA differed between groups (Table 2). At baseline, women exhibited more calcified plaques compared to men, while men had higher proportions of low-attenuating necrotic core and fibro-fatty plaque compared to women (Table 2). These sex differences in plaque composition were also observed at follow-up, where men displayed less high-attenuating fibrous and calcified plaques, and more low-attenuation plaques, including fibro-fatty plaques and necrotic core, compared to women (Table 2).

### 3.3 Sex differences in plaque progression

**Fig. 2:**
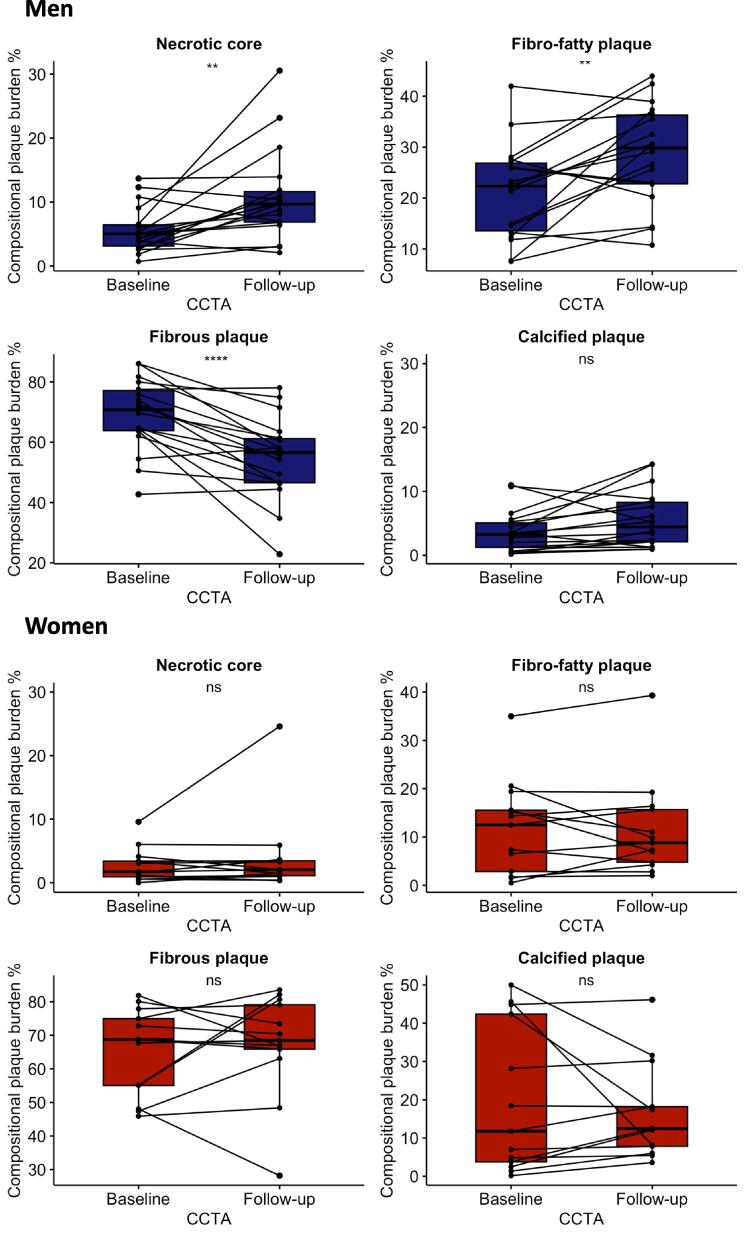
Change in compositional plaque burden in men and women at baseline and follow-up CCTA. The boxes show the medians and quartiles. ns: p > 0.05. *p ≤ 0.01. **p ≤ 0.0001.

Women displayed more stable plaque characteristics over time compared to men, with no significant change in plaque compositions between baseline and follow-up CCTA (Fig. 2). However, men had an increased number of segments with CAD (2.5 [1.0–4.0] vs. 3.0 [2.0–4.25] segments, p = 0.005), a higher percentage of necrotic core and fibro-fatty plaque compositions and decreased fibrous plaques at follow-up CCTA (Fig. 2). Although women consistently displayed a higher total plaque burden corrected for vessel volume across the serial CCTA examinations, no difference in the progression of total plaque burden between men and women was observed (−0.1 ± 1.6 % per year vs. −0.2 ± 1.8 % per year, p = 0.811, respectively).

**Fig. 3:**
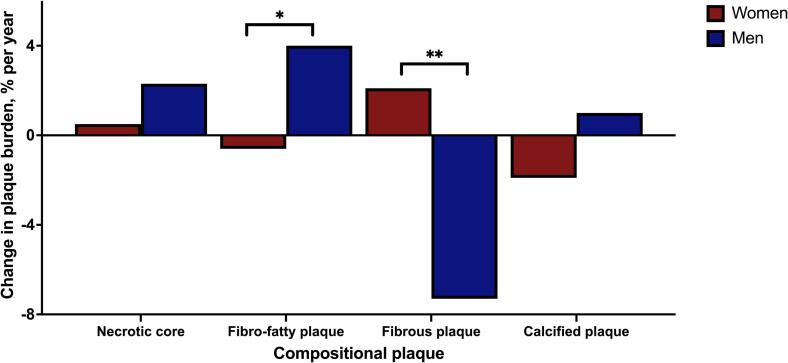
Annual change in plaque composition according to sex and adjusted for age. *p = 0.001 and age-adjusted p = 0.019. **p < 0.001 and age-adjusted p = 0.003.

The annual progression rate of low-attenuating plaque composition differed between sexes (Fig. 3). The progression of low-attenuating fibro-fatty plaques was faster in men compared to women, and men displayed a greater reduction in high-attenuating fibrous plaques compared to women even after age adjustment (Fig. 3). Although a trend towards faster progression of necrotic core in men and calcified plaques in women was observed, this did not reach statistical significance (Fig. 3).

## 4 Discussion

This study demonstrates that in patients with non-obstructive CAD undergoing serial CCTA, women had a greater plaque burden corrected for vessel volume with more calcified plaque compared to men, and further, displayed more stable plaque characteristics without any significant change in plaque composition over time. On the contrary, men exhibited more vulnerable low-attenuating plaque compositions, had a faster progression of low-attenuating fibro-fatty plaque, and a greater reduction of stable high-attenuating fibrous plaque compared to women, with an associated increase in diseased segments at follow-up. Progression of total plaque burden corrected for vessel volume did not differ between sexes. Even though women were older, age adjustment did not change the results.

Sex differences in the development and progression of coronary artery atherosclerotic plaques have previously been described. In general, among patients with stable chest pain, women are reported to have lower atherosclerotic plaque burden, less coronary artery calcification and more often non-obstructive CAD compared to their male counterparts [21], [22], [23]. However, women in our study undergoing clinically indicated serial CCTA, displayed an increased baseline and follow-up plaque burden and calcification compared to men, after accounting for sex disparities in vessel volume. Older age could account for the increased plaque burden seen among women in our study, as increasing age has been associated with higher total plaque burden and progression, driven by the calcified plaque component [24], [25]. This also supports the previously observed delay in atherosclerosis development in women [14]. However, age adjustment did not change the study results.

CV risk factor trajectories differ in women and men during the life span [26]. In this study, men tended to have a higher burden of CV risk factors compared to women, which may contribute to the increased progression of vulnerable plaque observed in men. Statin therapy has also been associated with increased coronary artery calcification as well as reduced progression of total plaque volume [5]. However, in the present study no difference in prevalence of statin therapy between men and women was observed at baseline or during follow-up.

Coronary artery plaque composition has been linked to plaque vulnerability for rupture and erosion, and acute CV events [1]. Vulnerable plaques can be detected non-invasively by CCTA as low-attenuation plaques [20]. Histopathologic sex differences in plaque transformation with plaque erosion and thrombus formation have been reported to be more prevalent in premenopausal women, and plaque rupture more prevalent in men and post-menopausal women [22]. In line with previous studies using serial CCTA, this study demonstrates that women had less vulnerable low-attenuating fibro-fatty plaques, while men displayed faster progression of vulnerable low-attenuating fibro-fatty plaques even after adjusting for age [11], [13]. These findings are important and may explain the observed increased CV risk in men, as both low-attenuation plaque and faster plaque progression by CCTA have been associated with adverse cardiac events [3], [27].

The progression of total plaque burden in our study did not differ between women and men over time, as also demonstrated in other studies using serial CCTA [11], [14]. However, El Mahdiui et al. previously demonstrated that women <55 years showed a faster regression of fibrous and non-calcified plaques compared to men, but this difference was not observed in women >55 years, perhaps due to the loss of the protective effects of estrogen [11]. Several protective effects of estrogen on the development and progression of atherosclerotic plaque have been suggested, including its effect on the physiology of the endothelial cells, smooth muscle cells of the arterial wall, cardiomyocytes, extracellular matrix deposition, inflammation, and coagulation [26], [28]. Women in our study, who were mostly post-menopausal, displayed an unaltered plaque composition over time. Despite slower plaque progression in women, the prevalence of coronary microvascular disease among patients with non-obstructive CAD is still more common in women compared to men [29].

Even though the progression of total plaque burden might be similar in women and men, the compositional age-adjusted plaque progression differs [14], highlighting the importance of quantitative plaque evaluation for refined risk assessment in non-obstructive CAD [30]. In clinical practice, identifying high-risk plaque phenotypes, such as vulnerable low-attenuation plaques and rapid plaque progression in patients with non-obstructive CAD and chronic coronary syndrome, could potentially contribute to the identification of patients in need of even more aggressive treatment and surveillance to improve prognosis.

### 4.1 Study limitations

There are several study limitations to be considered. First, this is an observational study with limited sample size. As a result, we are not able to assess any causal relationships and findings should be interpreted with caution. In addition, subgroup analysis in pre- and post-menopausal women are not possible due to lack of statistical power. Second, our study might be subject to selection bias as only patients with non-obstructive CAD undergoing serial CCTA examinations on clinical indication were included. Patients with non-obstructive CAD, severe symptoms and high CV risk may be more likely to be referred to invasive coronary angiography instead of repeated CCTA. Third, using semi-automated plaque analysis might result in measuring inaccuracies [20]. However, we demonstrated an excellent intra-observer reliability.

Despite these limitations, the study adds insight into the associations between sex and compositional plaque progression in non-obstructive CAD that may have clinical implications. However, our findings must be confirmed in large prospective trials. In addition, the association between compositional plaque progression and observed sex differences in cardiovascular events and mortality, as well as the effect of treatment on plaque progression must be clarified in clinical trials before implementation of serial CCTA and advanced compositional plaque analysis can be recommended in clinical practice guidelines. Integration of advanced compositional plaque volume and other plaque characteristics in clinical practice also depends on development of improved machine learning techniques to further increase the accuracy and availability of advanced plaque analysis.

## 5 Conclusion

In patients with chronic coronary syndrome and non-obstructive CAD, age-adjusted progression of compositional plaque differed between women and men undergoing clinically indicated serial CCTA. While women had a higher total plaque burden corrected for vessel volume and showed unaltered plaque composition over time, men had higher proportions of vulnerable low-attenuation plaques at both baseline and follow-up CCTA and displayed faster progression of vulnerable low-attenuating fibro-fatty plaques and a greater reduction of stable high-attenuating fibrous plaques compared to women. There was no difference in the progression of total plaque burden between women and men over time. Whether the observed sex differences in compositional plaque progression are associated with risk of acute CV events and thereby have implications for risk stratification, prevention, and treatment in non-obstructive CAD needs further investigation.

## CRediT authorship contribution statement

**Caroline Annette Berge Hondros:** . **Ingela Khan:** Formal analysis, Writing – review & editing. **Margrete Solvik:** Investigation, Writing – review & editing. **Silja Hanseth:** Investigation, Writing – review & editing. **Eva Kristine Ringdal Pedersen:** . **Siren Hovland:** Resources, Writing – review & editing. **Terje Hjalmar Larsen:** Resources, Supervision, Writing – review & editing. **Mai Tone Lønnebakken:** .

## Declaration of competing interest

The authors declare that they have no known competing financial interests or personal relationships that could have appeared to influence the work reported in this paper.
